# *Plasmodium* and intestinal parasite perturbations of the infected host’s inflammatory responses: a systematic review

**DOI:** 10.1186/s13071-018-2948-8

**Published:** 2018-07-03

**Authors:** Aminata Colle Lo, Babacar Faye, Ben Adu Gyan, Linda Eva Amoah

**Affiliations:** 1 Noguchi Memorial Institute for Medical Research, University of Ghana, Accra, Ghana; 2 University Cheikh Anta DIOP, Dakar, Senegal

## Abstract

Co-infection of malaria and intestinal parasites is widespread in sub-Saharan Africa and causes severe disease especially among the poorest populations. It has been shown that an intestinal parasite (helminth), mixed intestinal helminth or *Plasmodium* parasite infection in a human induces a wide range of cytokine responses, including anti-inflammatory, pro-inflammatory as well as regulatory cytokines. Although immunological interactions have been suggested to occur during a concurrent infection of helminths and *Plasmodium* parasites, different conclusions have been drawn on the influence this co-infection has on cytokine production. This review briefly discusses patterns of selected cytokine (IL-6, IL-8, IL-10, TNF-α and INF-γ) responses associated with infections caused by *Plasmodium*, intestinal parasites as well as a *Plasmodium*-helminth co-infection.

## Background

**Fig. 1:**
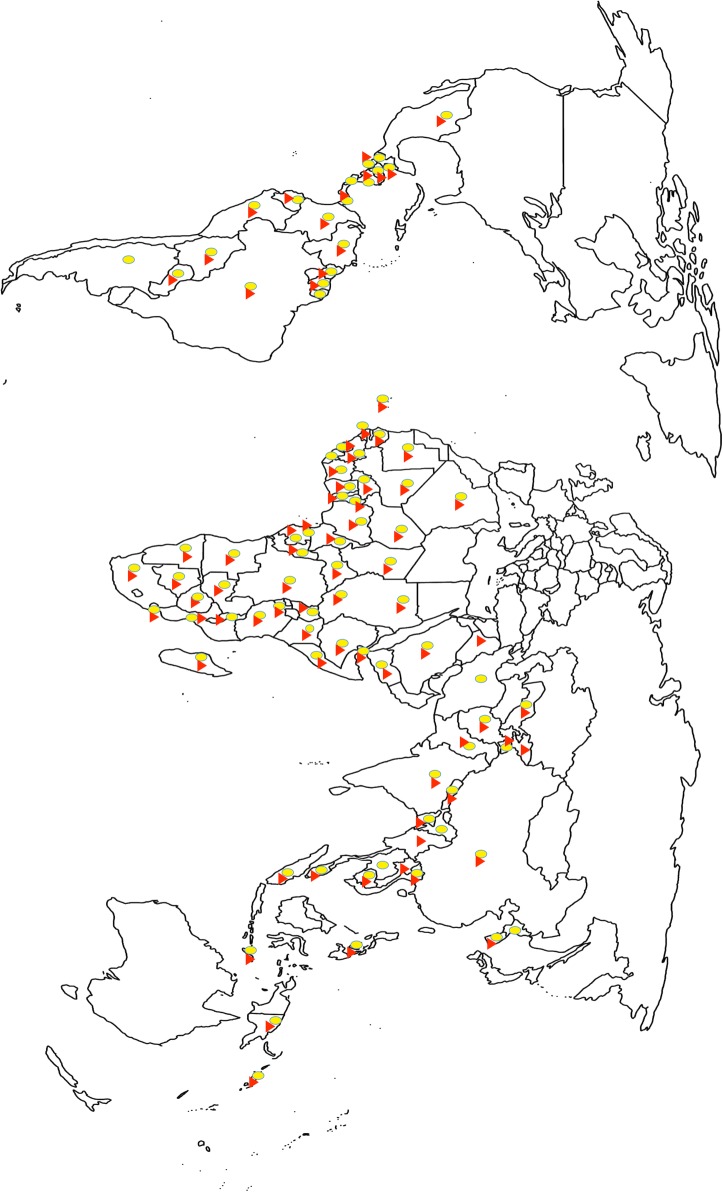
Distribution of people suspected to have malaria and children with soil-transmitted helminthiases. A map showing the global distribution of people in 2016 with suspected malaria (ovals) and children requiring helminth treatment (triangles). Countries with both an oval and a triangle are burdened with both diseases. The data for the soil-transmitted helminthiases was obtained from the Global Health Observatory (GHO) data [2] and the suspected malaria cases obtained from the 2017 WHO Malaria Report [1].

Malaria, a significant contributor to high childhood mortality, is endemic in the tropical regions as are pathogenic helminth infections. The distribution patterns of malaria parasites (*Plasmodium* spp.) and helminths coincide [1, 2], making malaria-intestinal parasite co-infections very common occurrences in most malaria endemic countries (Fig. 1, Table 1). In patients infected with *Plasmodium* spp., the interdependence between pro- and anti-inflammatory mediators of immunity can influence the survival of the *Plasmodium* parasite and subsequent development of disease [3]. In humans, helminth infections have strong immune-modulatory effects that could impact on other co-infecting parasites [4]. Additionally, helminth infections can cause anemia in the host. Anemia during a *Plasmodium* infection enhances gametocyte production [5], which can result in an increase in malaria transmission. Thus, an effective malaria control strategy should include critical knowledge of the impact helminth infections exert on *Plasmodium* parasite development and survival in the host.

**Table 1:** Prevalence of *Plasmodium*-helminth co-infections. A selection of malaria-helminth co-infection prevalence data obtained from studies conducted in Africa and South America

Country	Year	Prevalence (%)	Reference
Cameroon	2008	13.9	[127]
Cameroon	2014	22.1	[128]
Cameroon	2015	11.9	[129]
Cameroon	2016	11.6	[16]
Colombia	2012	24.5	[130]
Ethiopia	2009	23.6	[131]
Ethiopia	2010	39.6	[132]
Ethiopia	2012	19.4	[133]
Ethiopia	2012	5.2	[134]
Gabon	2010	15.0	[135]
Ghana	2009	31.0	[136]
Ghana	2009	30.7	[9]
Ghana	2011	30.5	[137]
Indonesia	2016	7.1	[138]
Ivory Coast	2012	24.7	[139]
Ivory Coast	2014	13.5	[140]
Kenya	2008	26.7	[141]
Kenya	2009	37.8	[142]
Kenya	2011	0.9	[143]
Kenya	2013	4.7	[144]
Kenya	2015	14.3	[145]
Malawi	2011	21.4	[146]
Nigeria	2011	4.3	[147]
Nigeria	2013	20.9	[148]
Nigeria	2013	42.9	[149]
Tanzania	2014	17.9	[150]
Tanzania	2017	26.4	[151]
Thailand	2010	19.0	[13]
Uganda	2005	54.8	[86]
Uganda	2008	15.5	[152]
Uganda	2010	15.5	[153]
Uganda	2011	9.3	[143]
Zambia	2012	44.3	[154]

It is evident from several human epidemiological surveys that have assessed variations in cytokine responses during parasitic infections that contradictory findings have been reported on responses identified during *Plasmodium*-helminth co-infections. For example, some studies on co-infections of *Plasmodium* and selected helminths such as *Schistosoma haematobium* and *Trichuris trichiura* have reported an increase in malaria parasite prevalence, parasite density, frequency and severity of disease [6–10]. Other studies have suggested that co-infection with *Plasmodium* and selected helminths including *Schistosoma mansoni* or *S. haematobium*, *Ascaris lumbricoides*, *Necator americanus* and *T. trichiura* provides protection against malaria [11–13]. Some studies, however, could not identify any effect of helminth (*S. haematobium* and *S. mansoni*) co-infection on the outcome of malaria [14, 15]. Two recent meta-analyses determined the overall odds of asymptomatic malaria to be slightly lower in uninfected children compared to children infected with soil-transmitted helminths (STH), including, *N. americanus*, *A. lumbricoides T. trichiura* or *S. mansoni* or *S. haematobium* [16, 17]. It has, however, been noted that immune responses to the different forms (eggs, adults and cercariae) of helminths differ, such as the differences noted in inflammatory responses to *S. mansoni* eggs and larvae [18]. As such immune responses during *Plasmodium-*helminth co-infections are naturally expected to differ, depending on the form (stage) of the infecting helminth (egg, larvae or adult). This then could account for the variations in results obtained in the reported studies. The risk of anemia has been suggested to be high in STH infections as is an increase in the prevalence of malaria during a *Plasmodium*-*Schistosoma* spp*.* co-infection [19]. During the 2-day life-cycle of the asexual *Plasmodium falciparum* parasite, some developmental life-stages (trophozoites and schizonts) are sequestered and evade immune recognition [20]. The 3 to 13 week helminth (varies for specific helminthes) life-cycle in the human similarly goes through various developmental stages, each eliciting variations in immune responses in the human host [21]. This makes the timing and order in which a parasitic mono-infection becomes a co-infection additional contributing factors that could account for the disparities in immune responses reported during *Plasmodium-*helminth co-infections.

## Cytokines associated with malaria infections

During a *Plasmodium* infection, T-cell mediated inflammatory responses contribute to reduced parasite density as well as the pathology of the disease [22, 23]. The mechanism by which the host controls parasite density *via* regulating inflammatory processes remains poorly understood [24], although a number of human and animal studies have suggested that the extent to which the host regulates the level and longevity of inflammatory responses generated against the parasite governs the efficient clearance of the parasite during the infection [25]. An optimum balance of cytokines is always needed at the different stages of the infection [26] to ensure effective parasite clearance. Pro-inflammatory responses such as IL-12, IFN-γ and TNF-α are Th1 cytokines, which are predominantly expressed during a *Plasmodium* infection in both humans and small rodents [27]. The Th1 cytokines including IL-6, IL-8, IL-12 and TNF-α are produced by a number of different cells including macrophages, dendritic cells and antigen presenting cells [28] are crucial to regulating parasite density at the beginning of a *P. falciparum* infection [29, 30].

### Systemic cytokines associated with human malaria

An acute *P. falciparum* infection is usually associated with an increase in pro-inflammatory responses, which subsequently cause an increase in the secretion of IFN-γ and TNF-α [31]. An early IFN-γ response is crucial in protecting against the development of the symptoms associated with severe malaria [32–34]. However, during a chronic *P. falciparum* infection, cytokines including IL-12, IFN-γ and TNF-α can induce adverse immunopathology if not well regulated [35]. The induction and expansion of regulatory T (Treg) cells are crucial during chronic disease [25, 36, 37] because Th2 cytokine cells can counteract Th1 responses.

A study conducted on the Dogon and the Fulani people of Mali, who live within the same environment but respond very differently to *P. falciparum* infections (Fulani are more resistant to malaria than the Dogon), found that IL-6, IL-8, IL-12 and IFN-γ levels in uninfected Fulani children were much higher than in uninfected Dogon children. The levels of IL-8 in Fulani infected with *P. falciparum* were significantly lower than an uninfected Fulani; however, the same was not observed among the Dogon people. They also identified IFN-γ as the only cytokine that was significantly higher in *P. falciparum*-infected Fulani children compared to matched Dogon children [38]. Stimulation of mononuclear cells from a Fulani with *Plasmodium* parasites was found to produce 10-times higher IFN-γ responses compared to similar cells from the Dogon [38].

TNF-α is predominantly secreted by activated macrophages and its pyrogenic properties are central to the immune response generated during a *Plasmodium* infection [39]*.* IL-6 has been suggested to be an essential component of the immune response during the acute phase [40] and in complicated *P. falciparum* malaria [41]. Elevated levels of IL-6 have also been associated with an increase in the incidence of clinical malaria [41]. This suggests that the IL-8 contribution to protection may be specific to the Fulani, especially as elevated levels of IL-8 have been reported in adults with severe malaria in Thailand [42]. Additional studies are needed to clarify the roles of IL-6 and IL-8 during *Plasmodium* infections.

Interleukin 10 in combination with other cytokines play an important role as immune regulators by neutralizing the Th1 effects associated with the more severe forms of *Plasmodium* infection [35]. Plasma levels of IL-10 in children with asymptomatic and mild malaria were found to correlate positively with the parasite load and to reduce significantly after the parasites in the peripheral blood were cleared [43]. Elevated levels of IL-10 with low TNF-α have been associated with mild malaria [43], whilst recovery from malaria (parasite clearance) has been associated with reduced levels of IL-10 [44]. Similarly, IL-10 levels in Zambian children under six years-old with severe malaria anemia were not significantly associated with protection [45]. The development of severe malaria anemia in African children may be a result of a lack of IL-10 production in response to high TNF-α concentrations [46]. Furthermore, increased IL-10 but not IL-12 production has been associated with increased parasite density in Mozambican children with both severe and uncomplicated malaria [47].

### Cytokines associated with murine malaria

Animal studies have been used to buttress some findings observed in *in vitro* and *in vivo* human studies relating to the use of cytokines in the control of malaria parasites. Murine models of malaria demonstrate a regulation of balance between IL-10 and inflammatory cytokines such as IFN-γ and TNF-α [48]. IL-10 production early in a rodent malaria infection has been found to prevent high parasite loads due to reduced Th1 responses [48] and the absence of regulatory cytokines during the later stages of the infection resulted in the development of adverse immuno-pathology [48]. Although pro-inflamatory responses involving cytokines including TNF-α, IFN-γ have been identified as contributors to the pathology of cerebral malaria in mice [49], early production of IFN-γ has been found to be crucial for preventing cerebral malaria [50].

In summary, human and murine models have both suggested IFN-γ to be essential for parasite clearance and reduced parasite multiplication rates [51–53]. However, regulated IFN-γ levels are required to avoid immune pathology [26, 39]. These characteristics of IFN-γ suggest it to be an ideal marker for tolerance to *Plasmodium* infections. Increased levels of IFN-γ, TNF-α, IL-12 and IL-10 are known to be associated with a reduced risk of malaria; however, those who become infected with malaria parasites have an elevated risk of symptomatic malaria [54]. These cytokines are also essential for inhibiting parasite development, stimulating parasite clearance and interacting with macrophages, which amongst other processes control infections through antibody-dependent and independent phagocytosis [55]. These subsequently resulted in the suppression of merozoite invasion into erythrocytes and subsequently fewer parasitized erythrocytes. More information on processes that occur in the immune system during a *Plasmodium* parasite infection can help develop malaria vaccines as well as help in designing other strategies for malaria control interventions such as intermittent preventive treatment (IPT) or seasonal malaria chemoprevention (SMC).

## Effects of intestinal parasite infections on cytokine profiles

Helminths survive by modulating the host’s immune system [17]. The initial immune response to intestinal helminths is usually Th1, which is then overtaken by Th2 during the course of the infection. Elevated Th2 response [56, 57] and the production of a regulatory network of immune responses [58, 59] are hallmarks of chronic helminthiasis that have the potential to impact the host’s immune response to other antigens [60]. The chronic immune activation caused by a helminth infection can alter T-cell memory responses and result in altered Th1 responses [18, 61, 62]. However, some intestinal helminth interactions with the host, such as host skin penetration by schistosome cercariae released from the intermediate host (a snail) induce strong Th1 responses. These responses make the host susceptible to inflammation characterized by the production of TNF-α together with other cytokines such as IL-1, IL-8, IFN-γ [63, 64]. TNF-α has been reported to enhance [65, 66] as well as decrease [67] the egg laying properties of female *S. mansoni* parasites. The role played by TNF-α in the metabolism of the adult *S. mansoni* parasite also remains controversial [68]. A comprehensive description of the processes involved in the TNF-α pathway in helminths is not readily available.

Although concurrent multiple (mixed) helminth infections are frequent occurrences, little is known about how concurrent parasite infections, such as *S. mansoni*, *S. haematobium* and *A. lumbricoides*, influence immune responses in a patient. There are only a few studies that have tried to understand the effect of multiple helminths and intestinal protozoan parasite co-infections on immune responses in children [69–71]. The IL-10 levels of children infected with mono or mixed helminths have been found to be similar to the levels in uninfected children; however anthelmintic treatment of the infected children resulted in a significant reduction in IL-10 levels compared to the uninfected children [70]. Similarly, when peripheral blood mononuclear cells (PBMCs) from adults co-infected with three parasites (filaria, hookworm and *Entamoeba histolytica*) were stimulated with helminth-specific antigens, they produced higher IL-10 levels compared with PBMCs from adults without the triple infection [72]. Filarial infection in Malian children aged between 11 and 17 years was found to have a higher *ex vivo* frequency of CD4+ cells producing IL-10 and IL-4 compared with those without the infection [73]. IL-10 can modulate Th1 responses by reducing pro-inflammatory cytokine, including TNF-α, IFN-γ and IL-12 responses [72] as well as enhancing immune suppression by preventing symptoms of inflammation [74, 75]. An increase in IL-10 levels may generate chronic disease pathogenesis. Co-infection with different helminths may counteract the IL-10 effect and the distinct cytokine response profiles generated may be used to define immunity as well as the severity of the resultant disease [68, 76]. A chronic co-infection of Brazilian children aged between 4 and 11 years with *A. lumbricoides* and *T. trichiura* was associated with an acute production of IL-10 in response to stimulation with *A. lumbricoides* antigen [77]. However, another study in young adults similarly co-infected with *A. lumbricoides* and *T. trichiura*, identified similar levels of IL-10 in the uninfected control, the mono- and poly-parasite infected groups [78]. The balance between Th1 and Th2 immune responses induced in a host during a multiple mixed intestinal parasite infection can hinder immune clearance of one of the co-infecting parasites over the other as has been suggested from a study on young children within independent or co-infections with *Giardia lamblia* and *Ascaris lumbricoides.* Higher levels of TNF-α was found in the co-infected children compared to matched children with only a *Giardia* infection and the IL-10/IFN-γ ratio in these co-infected children was higher than in uninfected children as well as children with only an *Ascaris* infection [79]. These results suggest that although the host most often is able to control infecting helminth load by initiating Th2 cytokine production, the presence of concurrent intestinal parasites can alter the cytokine responses and lead to persistence or a chronic infection of one of the infecting parasites.

## Cytokine profile during co-infection of *Plasmodium* and intestinal parasites

The precise impact of polyparasitism on the immunopathology of malaria is not known even though malaria co-infections with intestinal parasites in tropical regions are common [4]. Several studies conducted in Africa demonstrate that infection with STH and *Schistosoma* spp. may affect the immune response of a host to a malaria parasite infection and lead to increased susceptibility and disease severity [80–83]. However, helminthic infections have also been demonstrated to offer protection against severe malaria and anemia [84]. The variations reported in these study outcomes could be due to differences in the co-infecting helminth species, study design, study population and the level of immunity to *Plasmodium* parasites in the study population [85, 86].

*Ascaris* infections are more likely to afford protection against severe malaria while infections with hookworm or *Schistosoma* spp. are more likely to enhance the incidence of malaria [87]. The underlying immune responses generated against the different pathogens may have a strong effect on the development and pathological consequences of malaria. Diallo et al. [82] reported higher plasma levels of TNF-α and IFN-γ in *S. haematobium* and *P. falciparum* co-infected children compared with those infected with only *P. falciparum*. When blood from Ghanaian children with and without a *S. haematobium* infection was stimulated with *P. falciparum* antigens, a significantly higher level of IL-10 was recorded [17]. Similarly, IL-10 levels were significantly increased and INF-γ, IL-17 and TNF-α marked decreased when whole blood samples from asymptomatic malaria patients in Mali with filaria co-infections were stimulated with *P. falciparum* schizont lysate compared with the asymptomatic patients without filarial infection [73]. Children aged between 4 and 14 years with malaria and an asymptomatic *S. haematobium* infection were found to have significantly higher levels of IFN-γ but similar levels of IL-10 when compared to matched children without *S. haematobium* infection [88]. *Schistosoma haematobium* was found to protect against *P. falciparum* in children between 4 and 8 years-old [88]. IL-6 and IL-10 levels were found to correlate positively with acute malaria in children infected with *S. haematobium* who developed *P. falciparum* malaria when compared with *Schistosoma*-negative children who developed malaria. The effect was more pronounced in children aged between 9 and 14 years relative to those between 4 and 8 years [89]. Whilst a similar IL-10/IFN-γ ratio was observed in both the *Schistosoma*-positive and -negative children aged between 4 and 14 years who developed acute malaria, a significantly lower mean IL-10/TNF-α ratio was identified in *Schistosoma*-positive children aged between 9 and 14 years who developed acute malaria compared with matched *Schistosoma* negative children [89]. However, results from a study involving children co-infected with *Plasmodium* and *S. haematobium* did not demonstrate any influence of the co-infecting helminth on the immune response generated by the host against the *Plasmodium* parasite as a similar increase in both innate and adaptive immune responses was observed in the *P. falciparum*-infected and *P. falciparum*-*S. haematobium* co-infected children [90].

Helminth infections induce immuno-regulatory responses such as Th2 responses in the human host. These immune-regulatory responses can inhibit the ability of the host to mount an effective Th1 response [78], which also influences the immune response against the malaria parasite. When plasma levels of cytokines from individuals from the Amazon region of Brazil with either a *Plasmodium* infection or an intestinal parasite (*G. intestinalis*, *A. duodenale* and *S. stercoralis*) infection or concurrent *Plasmodium*-helminth (*G. intestinalis*, *A. duodenale* and *S. stercoralis*) co-infection were compared to uninfected individuals, the levels of TNF-α, IL-2, IL-10, IL-6 in the *Plasmodium* and *Plasmodium-*helminth co-infected group were similar, and both significantly higher than the uninfected group. These results corroborated the absence of additional stimulation of cytokine responses by the co-infecting helminth [91]. The median level of IFN-γ was, however, increased in all three (*Plasmodium*, intestinal parasite and *Plasmodium*-helminth co-infected) groups compared to the uninfected group [91]. In malaria, an efficient immunological balance prevents excessive multiplication of the parasite, which enables the malaria parasite survive in the host without the associated activation of inflammation. More studies are however needed to elucidate the immunological balance and the relationship between Th1, Th2 and chemokine responses that develop and moderate a *Plasmodium-*helminth co-infection as such information is very limited.

## Cytokine profile in pregnancy during a malaria and intestinal parasite co-infection

Pregnancy naturally causes alterations in immune responses and results in the dominance of Th2 immune responses. Increased IL-10 and a decrease in IFN-γ production have been associated with successful pregnancies in humans [92–94]. However, decreased IFN-γ and TNF-α production in animal models have been associated with poor pregnancy outcomes [93, 94]. These alterations in immune responses have been found to alter the susceptibility of pregnant women to some infectious diseases [95] including malaria [96, 97] and intestinal parasite infections [98, 99]. In humans, IFN-γ levels to schistosome egg and worm antigen has been found to decrease during pregnancy and high IL-10 levels [100–102] as well as IFN-γ, TNF-α, IL-10 and IL-6 [103] have been found to be associated with pregnancy associated malaria. Animal models have also shown a reduced schistosome specific IFN-γ production in pregnant mice [104]. Interleukin 10 responses to schistosome egg and worm antigen, however, are not altered by pregnancy in humans [105, 106]. The alterations in cytokine responses during the individual mono parasitic infections are likely to influence cytokine responses that occur during *Plasmodium*-helminth co-infections [98]. Although a number of studies have been conducted on pregnant women co-infected with *Plasmodium* and helminths, their main focus, which has been captured in a review by Mpairwe et al. [107], has been on anemia and birth outcomes, including infant birth weight, anemia, mortality and response to vaccines amongst others. More studies are needed to monitor changes in immune responses that occur across the three trimesters of pregnancy in women co-infected with these parasites as pregnancy makes women highly susceptible to mono and co-infections of these parasitic infections.

## Insights

We noticed the existence of several disparities relating to the association between various cytokine levels found in individuals co-infected with *P. falciparum* and different helminths. Diverse effects of a helminth infection on the susceptibility, severity and the pathology or the risk of cerebral malaria have been observed in several human studies conducted in Africa. These disparities could have arisen as a result of a number of different covariates, including the choice and nutritional status of the cohort(s) used in the study, the level of exposure, the infecting parasite species and the population and composition of microbial communities (microbiota) resident in the hosts’ intestines to name a few. In *P. falciparum* immunology, age is a strong confounder to antibody and cytokine analysis; however, in many of the studies monitoring the cytokine profiles of people with a malaria helminth co-infection, children with ages ranging from a few months with a low age range [91], to 19 years with a large age range [108] were used. TNF-α was found to be generally high in children aged between 11 and 12 years irrespective of their infection status. More consistent data could be obtained when the categories of age stratification is better described.

The definition of a *P. falciparum* and helminth infection differed between the studies, making the definition for a malaria helminth co-infection very different. A few studies defined malaria as *P. falciparum* positivity by real time PCR [89] and others used microscopy [89, 109]. Some studies defined a helminth infection as parasite positivity by real time PCR [91] or by observing eggs in stool and urine using microscopy [89, 91]. The sensitivity of the tests applied could interfere with sample grouping and subsequently influence the statistical analysis. In some instances, the cohort was asymptomatic for one [110] or both [91] the malaria and helminth infection. Other times, one infection was classified as symptomatic and sometimes chronic. A better understanding of cytokine responses generated during different presentations of an infection, such as during asymptomatic and symptomatic as well as high and low density infections, especially relating to malaria, could also help in providing a more stringent interpretation of and less disparity in the data obtained during *Plasmodium*-helminth co-infections under different infecting parasite conditions.

Nutrition directly impacts immunity as malnutrition and obesity have been found to result in immunodeficiency and reduced immunity, respectively [111], and indirectly influence the composition of resident microbiota in the host. Microbiota that colonize the human intestine have been found to regulate, including the modulation of T cell differentiation [112, 113] as well as be regulated by the human immune system [114]. Disruption of the homeostatic balance between the microbiota and the immune system can render the host susceptible to disease [115]. Animal studies have identified helminth infections to alter the microbiota composition in the infected animal and subsequently result in an increase in the adult worm population [116]. Luckily, the alteration in microbiota composition is reversible, with elimination of the adult helminth resulting in a restoration of the original microbiota composition [117] as well as immunity of the previously infected host. Controlling for nutritional status and resident microbiota population and composition within and between cohorts would be very challenging and as such would remain as an uncontrollable covariate.

These properties of the infection status all greatly influence the cytokine profile of the individual infections and likely would affect the cytokine profile of the co-infection. A list of cytokine responses associated with the innate and adaptive immune responses to *Plasmodium* and helminth infections and co-infections is shown in Table 2.

**Table 2:** Changes in cytokine profiles during parasitic infections. A selection of results obtained from selected *Plasmodium*, helminth and *Plasmodium*-helminth co-infection studies conducted in Africa and South America

Year	Country	Study population	Parasite	Cytokines	Reference
2016	Indonesia	All age groups	Helminths	Increased *in vitro* production of pro-inflammatory cytokines in response to *Pf* after treatment	[108]
2015	Gabon	Children	*Schistosoma haematobium*	Innate or adaptive immune response to *Pf* with or without *Schistosoma* co-infection was similar	[90]
2015	Kenya	Children	Polyparasite	IL-10 increased with *Schistosoma* infection but decreased in *Schistosoma*/filaria co-infections	[90]
				IL-6 increased with *Pf* infection but decreased in *Pf/*hookworm co-infection	[109]
				IL-6 and TNF-α levels were not affected by *Schistosoma* infection	[109]
2014	Nigeria	Children	*Schistosoma haematobium*	IL-10 did not change with *Schistosoma* infection but IFN- γ increased in older children	[110]
2014	Brazil	All age groups	*Giardia intestinalis*, *Ancylostoma duodenale*, *Strongiloides stercoralis*	TNF-α, IL-12, IL-10 and IL-6 were low but IFN-γ was high in co-infected people	[91]
2013	Ghana	All age groups	*Necator americanus* and *Giardia lamblia*	*Pf* exposure increased TNF-α, IL-17 and IL-7 production	[155]
2012	Mali	Children	*Schistosoma haematobium*	Higher Th1 cytokines in *Schistosoma*/*Pf* co-infections	[88]
2012	Mali	All age groups	Filaria	IL-12 low in filarial-positive, *in vitro* filarial-positive associated with IFN regulatory factor 1, no IL-12 in response to malaria antigen stimulation	[156]
2011	Mali	All age groups	Filaria	Filarial infection with asymptomatic malaria was associated with an increase in IL-4, IL-10 and IL-17A. PBMC stimulation with *Pf* antigen reduced IFN-γ, TNF-α and IL-17A in the filiarial infection	[73]
2011	Senegal	All age groups	*Schistosoma haematobium*	Higher IL-10 in the co-infected group	[157]
2010	Senegal	Children	*Schistosoma haematobium*	Schizont extract produced IL-10 only in co-infected group	[158]
2009	West Kenya	Children	*Schistosoma mansoni*	Memory T-regulators cells decreased in co-infected children	[159]

*Pf*, *Plasmodium falciparum*

Anthelmintic treatment (deworming) in humans has been suggested to enhance immune responsiveness to vaccines, especially to *Plasmodium* antigens in adults and children above 4 years old [118]. A major stumbling block to the validation of a malaria vaccine candidate is reduced immunogenicity. Currently, the only licensed malaria vaccine (RTS,S) is recommended to be administered as a four dose schedule in infants between 5 and 17 months old, which exhibited a 31.5% efficacy against severe malaria over a four year period [119]. Deworming children between 5 and 17 months is likely to result in enhanced immune responsiveness to the *Plasmodium* antigen (circumsporozoite protein, CSP) in RTS,S and subsequently increase the effectiveness of the vaccine. This increased immune responsiveness may also result in RTS,S efficacy in older children as well.

Experimental results obtained from the mice studies discussed in this review that have not been validated in human studies are to be taken as suggestive and to an extent that is probable but not definitive. These cautions are relevant because despite the genetic and physciological similarities between humans and mice [120, 121] and the fact that some studies have demonstrated similarities in observations made in both murine and human studies [122–124]. A number of studies have demonstrated disparities in results from murine and human studies such as fibrogenesis in mice being associated with a Th2 response but fibrogenesis caused by severe ‘hepatio-splenic’ schistosomiasis in humans associated with Th1 responses [125, 126].

## Conclusions

Several studies have provided evidence of immunological perturbations occurring during *Plasmodium* intestinal parasite co-infections; however, due to varying confounding factors, different mechanisms have been reported for the protection provided by different cytokines. A major source of variation in the results reported by the numerous studies could be the use of different cohorts and population (ethnic groups and age), stimulant (live parasite or recombinant antigen) and cell type (PBMC or plasma) as well as the different methods used to determine parasite prevalence and density.

Increased national efforts to reduce parasitic worm infections with frequent mass drug treatments may result in the modulation of the helminth induced cytokine response. It is thus imperative that the precise contribution intestinal parasites add on to immune responses generated during a malaria infection is fully understood. This will enable a better understanding of immune modulation when such a mass drug treatment is implemented. Increased efforts to obtain an effective malaria vaccine require a complete understanding of immune moderations generated during the co-infections of *Plasmodium* and intestinal worms.

## Abbreviations

IL: Interleukin
INF-γ: Interferon gamma
STH: Soil-transmitted helminths
TNF-α: Tumor necrosis factor
